# Interactions between peptidyl tRNA hydrolase homologs and the ribosomal release factor Mrf1 in *S. pombe* mitochondria

**DOI:** 10.1016/j.mito.2013.07.115

**Published:** 2013-11

**Authors:** Laurent Dujeancourt, Ricarda Richter, Zofia M. Chrzanowska-Lightowlers, Nathalie Bonnefoy, Christopher J. Herbert

**Affiliations:** a Centre de Génétique Moléculaire, UPR3404, FRC3115, Avenue de la Terrasse, 91198 Gif-sur-Yvette Cedex, France; b Wellcome Trust Centre for Mitochondrial Research, Institute for Ageing and Health, Newcastle University, Framlington Place, Newcastle upon Tyne NE2 4HH, UK

**Keywords:** Fission yeast, Mitochondria, Translation, Peptidyl tRNA hydrolase, Release factor, Mitochondrial ribosome tagging

## Abstract

Mitochondrial translation synthesizes key subunits of the respiratory complexes. In *Schizosaccharomyces pombe*, strains lacking Mrf1, the mitochondrial stop codon recognition factor, are viable, suggesting that other factors can play a role in translation termination. *S. pombe* contains four predicted peptidyl tRNA hydrolases, two of which (Pth3 and Pth4), have a GGQ motif that is conserved in class I release factors. We show that high dosage of Pth4 can compensate for the absence of Mrf1 and loss of Pth4 exacerbates the lack of Mrf1. Also Pth4 is a component of the mitochondrial ribosome, suggesting that it could help recycling stalled ribosomes.

## Highlights

• In *S. pombe* the peptidyl tRNA hydrolases Pth3 and Pth4 are mitochondrial proteins.
• Pth3 and Pth4 are associated with the mitochondrial ribosome and the large subunit.
• Deletion of *pth4* and *mrf1*, encoding the mitochondrial release factor, is co-lethal.
• Over-expression of *pth4* compensates for the deletion of *mrf1*.
• Pth4 can act as a release factor in *S. pombe* mitochondria.

## 1 Introduction

Mitochondria are essential organelles that are found in almost all eukaryotic cells, it is generally accepted that they arose from the endosymbiosis of α proteobacteria, closely related to *Rickettsia*, into a eukaryotic ancestor (Emelyanov, 2003). Their main function is to provide the cell with energy in the form of ATP, via oxidative phosphorylation (OXPHOS), a process completed by multi-protein complexes located in the inner mitochondrial membrane. Mitochondria contain their own genome, which encodes a small number of key OXPHOS subunits of the respiratory complexes, and most or all, of the RNAs needed for mitochondrial translation. The vast majority of the proteins involved in mitochondrial translation are of nuclear origin; thus both the OXPHOS complexes and the mitochondrial translation machinery are of dual genetic origin.

Due to the endosymbiotic origin of mitochondria, many aspects of translation resemble the bacterial system; however, there are some differences, e.g. variations in the genetic code, the absence of a Shine-Dalgarno type ribosome binding site (sometimes replaced by specific translational activators), and a smaller set of tRNAs and translation factors (reviewed in Herrmann et al., 2013).

Another notable difference is that although there is some limited transcriptional control, at least in humans where transcript levels do not always reflect changes in mtDNA abundance, transcription is not the principal point of regulation in mitochondria. For example, in *Schizosaccharomyces pombe* Atp9 and Apt6 are encoded on the same polycistronic primary transcript but there is a ten-fold difference in the levels of the proteins (Deshpande and Patel, 2012). Thus the post-transcriptional steps of mitochondrial RNA metabolism and especially translation are important control points of mitochondrial gene expression. Consequently, mitochondrial translation defects are a frequent cause of human diseases, both because this is a crucial step in respiratory complex biogenesis and because there are many mitochondrial and nuclear genes involved in the mechanism and regulation of translation. Mutations causing mitochondrial diseases have been identified in mitochondrial genes encoding ribosomal RNAs (rRNA) and transfer RNAs (tRNA), and also in a number of nuclear genes encoding mitochondrial ribosomal proteins (MRPs), aminoacyl tRNA synthetases, tRNA modification enzymes, and translation factors (Ylikallio and Suomalainen, 2012). Among the general translation factors, disease causing mutations have been found in elongation factors, such as mtEF-Tu, mtEF-Ts and mtEFG1, and more recently a mutation was identified in C12orf65, a predicted peptidyl tRNA hydrolase thought to act in translation termination and thus tRNA recycling (Antonicka et al., 2010).

Peptide release from the ribosome is an essential part of the normal termination of translation, but it is also needed to unblock stalled ribosomes, for example when translation has been initiated on a 3′ truncated mRNA. It is clear that the cell has evolved a variety of release factors and mechanisms to deal with these different situations. Typically, eubacteria contain two class I release factors, RF1 and RF2, that between them are able to recognize the three stop codons (UAA, UAG and UGA), as well as a class II release factor, RF3, that hydrolyzes GTP to stimulate the removal of RF1 and RF2 from the ribosome and initiate ribosome recycling (for review Duarte et al., 2012). In addition, bacteria like *Escherichia coli* contain at least three distinct systems to process stalled ribosomes: the tmRNA encoded by *ssrA* that initiates *trans* translation leading to termination, the peptidyl tRNA hydrolases Pth and YaeJ and finally ArfA, which recruits RF2 to stalled ribosomes (Chadani et al., 2011, Chadani et al., 2012, Singh and Varshney, 2004).

The situation appears to be simpler in mitochondria, for example there is only a single class I mitochondrial release factor (Mrf1 in yeast, mtRF1a in humans) recognizing all yeast and human mitochondrial stop codons (UAA and UAG) (Pel et al., 1992, Soleimanpour-Lichaei et al., 2007, Temperley et al., 2010). To unblock stalled ribosomes mitochondria appear only to have peptidyl tRNA hydrolases (Antonicka et al., 2010, Richter et al., 2010), although recently mtRF1, a sequence homolog of mtRF1a, has also been proposed to play a role in this process (Huynen et al., 2012).

The yeast *S. pombe* shares many characteristics with human cells and is a pertinent unicellular model to study the relationships between mitochondrial translation termination factors and the Pth proteins. First *S. pombe* is a *petite*-negative yeast, dependent upon mitochondrial function (Bulder, 1964). It has a compact mtDNA, like human organelles, and consequently *S. pombe* mitochondrial mRNAs have very short 3′ UTR extensions, again similar to human mitochondrial mRNAs. In addition *S. pombe* uses a set of mitochondrial translation factors very similar to that of human mitochondria (Chiron et al., 2005). Among these, the ribosome recycling factor Rrf1 and the stop codon recognition factor Mrf1 can be replaced by their human homologs (Rorbach et al., 2008, Soleimanpour-Lichaei et al., 2007). Finally, neither the deletion of the *mrf1* gene in *S. pombe*, nor the depletion of the human gene leads to a complete block in mitochondrial protein synthesis, suggesting that other proteins with an overlapping function exist.

We have searched for predicted peptidyl tRNA hydrolases from *S. pombe* and found Pth3 and Pth4, which are sequence homologs for the human proteins C12orf65 and ICT1 respectively. In this paper, we have investigated the relationships between the *S. pombe pth* genes and *mrf1* and we find that *pth4* plays an overlapping role with *mrf1*.

## 2 Materials and methods

### 2.1 Strains, plasmids, media and genetic methods

All the strains used in this study are described in Table 1 and were grown at 28 °C or 36 °C as indicated. Plasmids used or constructed during this work were derivatives of pGEM-T-easy (Promega), pDUAL-FFH1, pDUAL-YFH1 (Matsuyama et al., 2004), pTG1754/Not (Bonnefoy et al., 1996) and pSC49 (a *leu1* version of pTG1754, S. Chiron unpublished). Genes cloned in pDUAL-FFH1 will give rise to proteins that are tagged FLAG_2_His_6_. The human *ICT1* and *C12orf65* ORFs lacking the start codon were cloned into pSC49 fused to the *Neurospora crassa* F0-ATPase subunit 9 presequence and a C-terminal FLAG tag was added (Rojo et al., 1995). Media and genetic methods were as described in Bonnefoy et al., 1996, Bonnefoy et al., 2000. *S. pombe* asci were microdissected directly from the mixture of haploid, diploid and sporulating cells.

**Table 1:** *S. pombe* strains used in this work.

Strain name	Genotype	Reference
NB205-6A	*h− ade6*-*M216 ura4*-*D18 his3*∆ *leu1*-*32*	Chiron et al. (2005)
NB34-21A	*h*− *ade6*-*M216 ura4*-*D18 his3*∆ *leu1*-*32 ptp1*-*1*	Chiron et al. (2005)
NBp9-725	*h*+ *ade6*-*M216 leu1*-*32*	This work
NB204-14B	*h*− *ade6M*-*210*, *his3*∆	This work
CHP056	*h*+ *ade6*-*M216 leu1*-*32 mrp4*-*His_7_::kan^R^*	This work
CHP056-2A	*h*− *ade6M*-*210 his3*∆ *mrp4*-*His_7_::kan^R^*	This work
CHP060	*h*+ *ade6*-*M216 leu1*-*32 mrpl12*-*His_7_::kan^R^*	This work
CHP060-2D	*h*+ *ade6 leu1*-*32 mrp4*-*His_7_::kan^R^ mrpl12*-*His_7_::kan^R^*	This work
LD63-1	*h*+ *ade6 mrp4*-*His_7_::kan^R^ mrpl12*-*His_7_::kan^R^ leu1::pth3*-*FLAG_2_*-*His_6_*	This work
LD64-1	*h*+ *ade6 mrp4*-*His_7_::kan^R^ mrpl12*-*His_7_::kan^R^ leu1::pth4*-*FLAG_2_*-*His_6_*	This work
LD65-1	*h*+ *ade6 mrp4*-*His_7_::kan^R^ mrpl12*-*His_7_::kan^R^ leu1::mrf1*-*FLAG_2_*-*His_6_*	This work
KV8-7	*h*− *ade6*-*M216 ura4*-*D18 his3*∆ *leu1*-*32* ∆*pth3::kan^R^*	This work
NB338-1D	*h*+ *ura4*-*D18 leu1*-*32* ∆*pth3::kan^R^*	This work
MG49-17	*h*− *ade6*-*M216 his3*∆ *leu1*-*32 ura4*-*D18* ∆*pth4::kan^R^*	This work
MG50-12	*h*− *ade6*-*M216 leu1*-*32 ura4 ptp1*-*1* ∆*pth4::kan^R^*	This work
MG50-24	*h*− *ade6*-*M216 leu1*-*32 ura4 ptp1*-*1* ∆*pth4::kan^R^*	This work
LD1-2A	*h? ade6 ura4*-*D18 leu1*-*32* ∆*pth3::kan^R^*	This work
LD1-2B	*h? ura4*-*D18 his3*∆ *leu1*-*32* ∆*pth3::kan^R^* ∆*pth4::kan^R^*	This work
LD1-2C	*h? ade6 ura4*-*D18 leu1*-*32*	This work
LD1-2D	*h? ura4*-*D18 his3*∆ *leu1*-*32* ∆*pth4::kan^R^*	This work
NB329-1	*h*− *ade6*-*M216 his3*∆ *leu1*-*32 ura4*-*D18* ∆*mrf1::kan^R^*	Soleimanpour-Lichaei et al. (2007)
NB349-6D	*h*+ *leu1*-*32 his3*∆ ∆*mrf1::kan^R^*	This work
NB334-5C	*h*+ *ade6 ura4 leu1*-*32 ptp1*-*1* ∆*mrf1::kan^R^*	This work
LD5-4A	*h? leu1*-*32 his3*∆ ∆*pth3::kan^R^* ∆*mrf1::kan^R^*	This work
LD7-1B	*h? ura4 leu1-32* ∆*pth4::kan^R^* ∆*mrf1::kan^R^ ptp1*-*1*	This work
LD7-3B	*h? ura4 leu1*-*32* ∆*pth4::kan^R^* ∆*mrf1::kan^R^ ptp1*-*1*	This work
LD7-4A	*h? ura4 leu1*-*32* ∆*mrf1::kan^R^ ptp1*-*1*	This work
LD7-4B	*h? ade-M216 ura4 leu1-32 ptp1-1*	This work
LD7-4C	*h? ade-M216 ura4 leu1-32* ∆*pth4::kan^R^ ptp1-1*	This work
LD7-4D	*h? ura4 leu1-32 pth4::kan^R^* ∆*mrf1::kan^R^ ptp1-1*	This work
LD8-9A	*h? ura4 leu1-32 pth4::kan^R^* ∆*mrf1::kan^R^ ptp1-1*	This work
CHP079-6C	*h? ade6M mrps4-His_7_::kan^R^ mrpl12-His_7_::kan^R^* ∆*mrf1::kan^R^ leu1::pth4-FLAG_2_-His_6_*	This work

All strains contain 3 mitochondrial introns.

HEK293-Flp-In™T-REx™ cells (HEK293T) were from Invitrogen and were grown in Dulbecco's modified Eagle's medium with pyruvate and L-glutamine, supplemented with 1 × non-essential amino acids, 50 μg/ml uridine and 10% fetal bovine serum; untransfected cells were routinely cultured with 10 μg/ml blasticidin and 100 μg/ml zeocin. Cells were transfected at ~ 30% confluency as described in Rorbach et al. (2008).

### 2.2 *S. pombe* transformation

*S. pombe* cells were transformed either by a chemical method or by electroporation. The lithium acetate technique (Okazaki et al., 1990) was improved by (1) using single stranded salmon sperm DNA as carrier, (2) regenerating cells in complete liquid medium overnight, and (3) plating onto 5% glucose selective medium as described in Chiron et al. (2007). The electroporation protocol was based on several published procedures (Suga and Hatakeyama, 2001, Suga and Hatakeyama, 2009, Suga et al., 2000, Suga et al., 2004). Cells were grown in YNB from Difco with 2% glucose and supplements at 150 μg/ml to a density of about 1 × 10^7^ cells/ml. Cells were harvested by centrifugation at 4500 rpm for 5 min and resuspended in 0.1 volumes of 0.6 M sorbitol, 25 mM DTT, and 20 mM HEPES pH 7.0, incubated at 30 °C for 15 min and washed 3 times with 30 ml of ice cold 1 M sorbitol. The final cell pellet was resuspended at 10 × 10^9^ cells/ml in 1 M sorbitol. At this point aliquots of 50 μl were used directly for electroporation, or 50 μl aliquots were frozen and stored at − 80 °C. For transformation, aliquots of frozen cells were rapidly thawed in a water bath at 30 °C, centrifuged for 1 min at 5000 rpm, resuspended in 1 ml of ice cold 1 M sorbitol, centrifuged for 1 min at 5000 rpm and resuspended in ice cold 1 M sorbitol to give 50 μl. Up to 5 μl of DNA in H_2_O was added to the cell suspension. Immediately after electroporation 1 ml of 2 M sorbitol, 25 mM HEPES pH 7.0 was added and the mixture was incubated at 30 °C for 10 min, after which 0.2 ml aliquots were plated directly on selective medium. When constructing gene replacements and gene fusions using an antibiotic resistance marker it is preferable to allow 3–5 h of growth in rich glucose medium before plating on the selective medium. With 10 ng of plasmid DNA the transformation efficiency is routinely 1–2 × 10^4^ transformants per μg of DNA.

### 2.3 Construction of gene deletions in *S. pombe*

*S. pombe* gene deletions were constructed by the PCR method (Wach, 1996) using pFA6a-kanMX4 (carrying the *kan^R^* gene that confers G418 resistance, Wach et al., 1994). PCR fragments containing the *kan^R^* gene were generated with hybrid oligonucleotides containing 75 to 80 bases of homology with the recipient *locus* on both sides of the gene of interest and transformed into NB205-6A or NB34-21A as described in Chiron et al. (2007). [Kan^R^] transformants able to grow in the presence of the drug G418 were streaked again on selective medium, and the genomic DNA of single colonies was extracted (Hoffman and Winston, 1987) and analyzed by PCR to look both for the correct insertion of the deletion cassette and the absence of the wild type sequences. Colonies carrying the deletion were back-crossed to a wild type strain to verify the co-segregation of the G418 resistance with the gene deletion.

### 2.4 Construction of double mutants

To construct the various double mutants, single mutants were first crossed with a wild type to isolate spores of the opposite mating type: NB349-6D was an *h*+ spore from the ∆*mrf1* strain NB329 (Soleimanpour-Lichaei et al., 2007), NB334-5C was an *h*+ spore from the ∆*mrf1 ptp1*-*1* strain NB330 and NB338-1D was an *h*+ spore from the ∆*pth3* strain KV8-7. Then, NB349-6D was crossed with KV8-7 to generate the LD5-4A ∆*mrf1* ∆*pth3* double mutant, NB334-5C was crossed with MG50-12 and MG50-24 to generate the *ptp1*-*1* ∆*mrf1* ∆*pth4* strains LD4-3B, 4C, 4D and LD8-9A respectively, and NB338-1D was then crossed with MG49-17 to generate the LD1-2B ∆*pth3* ∆*pth4* double mutant.

### 2.5 Epitope tagging of the mitochondrial ribosomal protein genes

The genes encoding proteins from the small (Mrp4) or large (MrpL12) ribosomal subunits were epitope tagged at their chromosomal locus using a PCR strategy similar to that of the gene deletion (Wach, 1996), except that the forward primer contained an in-frame His_7_ tag coding sequence and pFA6a-13Myc-kamMX6 was used as the template in order to provide the terminator sequences (Petracek and Longtine, 2002). The initial gene fusions were made in NBP9-725 by integrative transformation of PCR fragments. The constructions were verified by PCR amplification of the 5′ section of the gene and the His_7_ tag and sequencing. The expression of the tagged protein was then verified by western blotting of whole cell extracts. CHP056 (producing Mrp4-His_7_), was crossed to NB204-14B and sporulated to give CHP056-2A, which has the opposite mating type; this was crossed to CHP060 (producing MrpL12-His_7_) and sporulated to give the doubled tagged strain CHP060-2D.

### 2.6 Integration of FLAG versions of the pth and mrf1 genes

Plasmids containing the tagged *S. pombe pth3*, *pth4* and *mrf1* genes under the control of the *nmt1* promoter (Matsuyama et al., 2006) were purchased from the RIKEN consortium and tested for their ability to complement the corresponding mutants or the double ∆*pth3* ∆*pth4* mutant by transformation and selection for the *ura4* marker to maintain the plasmid in its replicative form. The tagged genes Pth3-YFP-FLAG-His_6_ and Pth4-YFP-FLAG-His_6_ were able to complement the respective deletions but the RIKEN construct producing the Mrf1-YFP-FLAG-His_6_ protein carried several mutations and did not complement. To circumvent this problem, the Mrf1-YFP-FLAG-His_6_ plasmid was reconstructed by gap-repair, after PCR amplification of the *mrf1* ORF from genomic DNA. The complementing plasmids were cut by *Not*I and transformed into the corresponding ∆*pth3*, ∆*pth4* or ∆*mrf1* mutants to integrate the tagged version into the *leu1 locus* (see Matsuyama et al., 2004), or in the CHP60-2D strain containing His_7_ tagged versions of Mrp4 and MrpL12.

### 2.7 Purification of mitochondria, alkali treatment, immunoprecipitation and western blotting

Mitochondria were purified from *S. pombe* cells grown in complete glucose medium as described previously (Chiron et al., 2007). Alkali carbonate extraction to separate membrane and soluble mitochondrial fractions were performed on purified mitochondria as described in Lemaire and Dujardin (2008). Mitochondria were purified from HEK293T cells as described in Rorbach et al. (2008). For immunoprecipitation purified mitochondria were treated with DNase 1 and proteinase K to minimize unspecific contamination from cytosolic proteins, lysed and treated as described in Rorbach et al. (2008). Protein samples were separated on 10 or 12% SDS-PAGE before western blotting. Primary antibodies were: anti-*Saccharomyces cerevisiae* Arg8: 1/4 000 (Steele et al., 1996); anti-*S. pombe* Cox2, 1/2 500 (Gaisne and Bonnefoy, 2006); anti-FLAG, 1/1 000 (Sigma F185); anti-His 1/5 000 (Genscript); anti-MRPL3, 1/2 000, anti-MRPL12 1/1 000, anti-DAP3 1/1 000 (Abcam); anti-MRPS18B 1/4 000, and anti-ICT1 1/800 (Proteintech Group). Secondary antibodies were diluted 1/10 000.

### 2.8 Ribosome analysis

For *S. pombe*, cells were grown to an OD_600_ of 1–1.5 in complete or minimal glucose medium as required; the cells were then washed and resuspended in 2 × the pellet volume of lysis buffer (1% Triton X-100, 20 mM Hepes pH7.6, 40 mM KCl, 1 mM PMSF, 1 × protease inhibitors (Roche) and either 50 mM EDTA or 50 mM MgCl_2_), then broken under freezing conditions using a French Press. The cell extracts were allowed to thaw on ice and then clarified by centrifugation at 15,000 rpm and 4 °C for 10 min. The extracts (10 OD_260_ units) were layered onto 10 to 45% sucrose gradients in lysis buffer (25 mM KCl) containing either EDTA or MgCl_2_ and centrifuged at 39,000 rpm and 4 °C for 3.5 h in a SW41 rotor (Beckman). Gradient analysis was performed using an Isco Foxy R1 fractionator and continuously monitored at 254 nm. Typically, 30 fractions were collected. For subsequent western blot analysis the samples were treated as described in Daugeron et al. (2011).

For the HEK293T cell line, cells were harvested and washed once with PBS, resuspended in lysis buffer (50 mM Tris–HCl pH 7.5, 150 mM NaCl, 10 mM MgCl_2_, 1% Triton X-100, 1 mM PMSF and Roche EDTA free protease inhibitor cocktail), 50 μl of cold lysis buffer for 10 mg of wet cell pellet. Samples were incubated on a rotating wheel for 30 min at 4 °C prior to centrifugation at 12,000 *g* at 4 °C for 10 min. For the ribosome analysis, 700 μg of cleared cell lysate was separated on 10–30% isokinetic sucrose gradient.

### 2.9 Southern blot analyses

Total DNAs from cells grown in complete glucose medium were extracted as described previously (Hoffman and Winston, 1987) and electrophoresed on a 1% agarose gel before transfer onto Hybond-C extra membranes. The blots were hybridized overnight at 65 °C under standard saline conditions and after washing, were exposed for a few hours, or up to 2 weeks. The probes used were the complete *S. pombe* mtDNA cloned in pBR322 (del Giudice et al., 1983) labeled by nick-translation with dATP^32^ (Amersham) and the 1.8 kb *Hin*DIII fragment of *ura4* (Grimm et al., 1988) labeled with dCTP^32^ using a random priming kit (Invitrogen).

### 2.10 ^35^S labeling of mitochondrial proteins

*S. pombe* cells were grown to early exponential phase in minimal 5% raffinose medium containing 0.1% glucose. Mitochondrial proteins were labeled at 30 °C by a 3 hour incubation of whole cells with ^35^S methionine and cysteine (Bioactif-Hartmann) in the presence of 10 mg/ml cycloheximide (5 mg per reaction) that specifically blocks cytosolic translation. Proteins were extracted as described in Gouget et al. (2008) and samples were separated on 16% acrylamide–0.5% bisacrylamide SDS gels. After drying the gel was exposed to a film for one day or up to several weeks at − 70 °C, or to a phosphorimager screen at room temperature.

### 2.11 Cytochrome spectra

Low temperature cytochrome spectra of *S. pombe* frozen cell pastes were recorded using a Cary 400 spectrophotometer after addition of sodium dithionite to fully reduce the cytochromes (Claisse et al., 1970). The absorption maxima were 603, 560, 554 and 548 nm for cytochromes *aa3*, *b*, *c1* and *c* respectively. The *S. pombe* cytochrome *c* peak always shows a 544 nm shoulder that disappears in a cytochrome *c* mutant (N. Bonnefoy, data not shown).

## 3 Results

### 3.1 Localization of Mrf1, Pth3 and Pth4

**Fig. 1:**
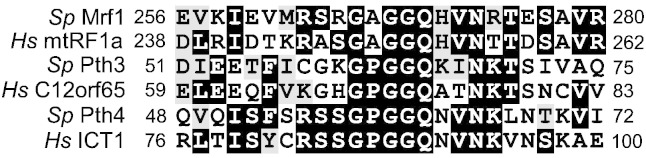
Multiple alignment of part of Mrf1 from *S. pombe* and mtRF1a from humans with the *S. pombe* Pth3 and Pth4 proteins and their human orthologs, C12orf65 and ICT1. The alignment was made using the full-length protein sequences and the ClustalW and Boxshade programs from the http://www.ch.embnet.org server as for Fig. S1, but only the region containing the GGQ motif is shown. Pth3: Spbc1105.18c; Pth4: Spac589.11.

In an attempt to further our understanding of the termination of mitochondrial translation in *S. pombe*, we decided to investigate the role of the mitochondrial Pth proteins and their possible interaction with the mitochondrial release factor, Mrf1. Pth proteins are ubiquitous in nature and are divided into several families, Pth1 are found in bacteria (Brun et al., 1971) and Pth2 are found in archaea (Rosas-Sandoval et al., 2002). Eukaryotes contain two other Pth proteins that have been given various names (Pth3 and Pth4 in yeasts) and are related to the mitochondrial release factors. Crystallographic studies of the eubacterial release factors, RF1 and RF2, in complex with the 70S ribosome show that the glutamine residue in a conserved GGQ motif is positioned in such a way that it can contribute directly to the hydrolysis of the peptidyl-tRNA bond (Laurberg et al., 2008 and Weixlbaumer et al., 2008). Fig. 1 shows an alignment of this region of Mrf1, Pth3 and Pth4 from *S. pombe* and the equivalent proteins from humans; it is clear that the GGQ motif is conserved in all these proteins (a full alignment of these three proteins from *S. cerevisiae*, *S. pombe* and humans is shown in Fig. S1). The mechanism of action of the Pth1 and Pth2 families is different from that of the mitochondrial release factor family and do not depend on a GGQ motif; we have deleted *pth1* and *pth2* and been unable to detect any respiratory phenotype (data not shown). These results and the sequence comparisons led us to focus our study on Pth3, Pth4 and Mrf1.

**Fig. 2:**
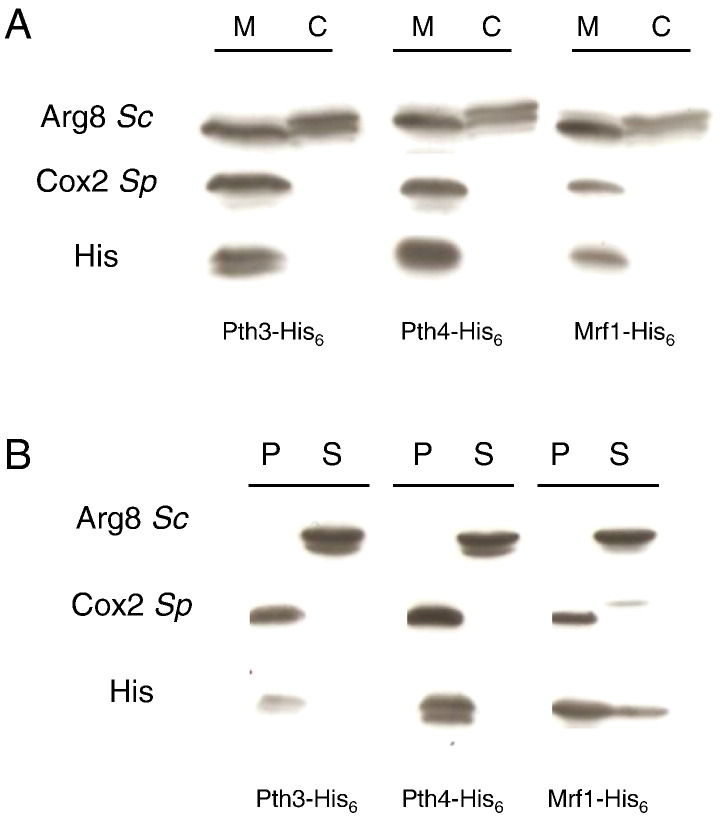
Mitochondrial localization and membrane association of Pth3, Pth4 and Mrf1. Mitochondria were purified from cells engineered to produce His_6_-tagged versions of Pth3, Pth4 or Mrf1 (LD63-1, LD64-1 and LD65-1 respectively) at their chromosomal *loci*. A. The post-mitochondrial supernatant corresponding to the cytosolic fraction (C) and purified mitochondria (M) were loaded on a 15% polyacrylamide gel and analyzed by western blotting with antibodies recognizing the His_6_-tag, the *S. pombe* Cox2 protein and the *S. cerevisiae* Arg8 protein. In *S. pombe* anti-Arg8 detects both Arg1, the *S. pombe* mitochondrial Arg8 homolog, and an unknown cytosolic protein. An equal amount of protein (50 μg) was loaded in each lane. B. Purified mitochondria were alkali-treated to separate the soluble (S) and membrane fraction (P), the total protein in each fraction was loaded on a 15% polyacrylamide gel and analyzed by western blot as in panel A.

First we decided to determine the localization of the three proteins Mrf1, Pth3 and Pth4; to do this strains carrying triple tagged versions of the corresponding genes under the control of the thiamine repressible *nmt1* promoter were constructed as described in the Materials and methods. Mitochondria were purified from these strains by cell fractionation and the mitochondria and post-mitochondrial supernatants were analyzed by western blot. The results, in Fig. 2A, show that all three proteins were only found in the mitochondrial fraction. To further refine the localizations, purified mitochondria were treated with alkaline carbonate, which will solubilize proteins that are loosely attached to the membrane. Fig. 2B shows that like the integral membrane protein Cox2, Pth3 and Pth4 were found uniquely in the membrane fraction, whereas Mrf1 partitioned between the membrane and soluble fraction. Thus Mrf1, Pth3 and Pth4 are all mitochondrial proteins, more-or-less tightly associated with the inner mitochondrial membrane. However, it is not possible to know if this membrane association is direct, or via their participation in a protein complex that is membrane associated, possibly the mitochondrial ribosome.

### 3.2 Deletion of pth3 and pth4

**Fig. 3:**
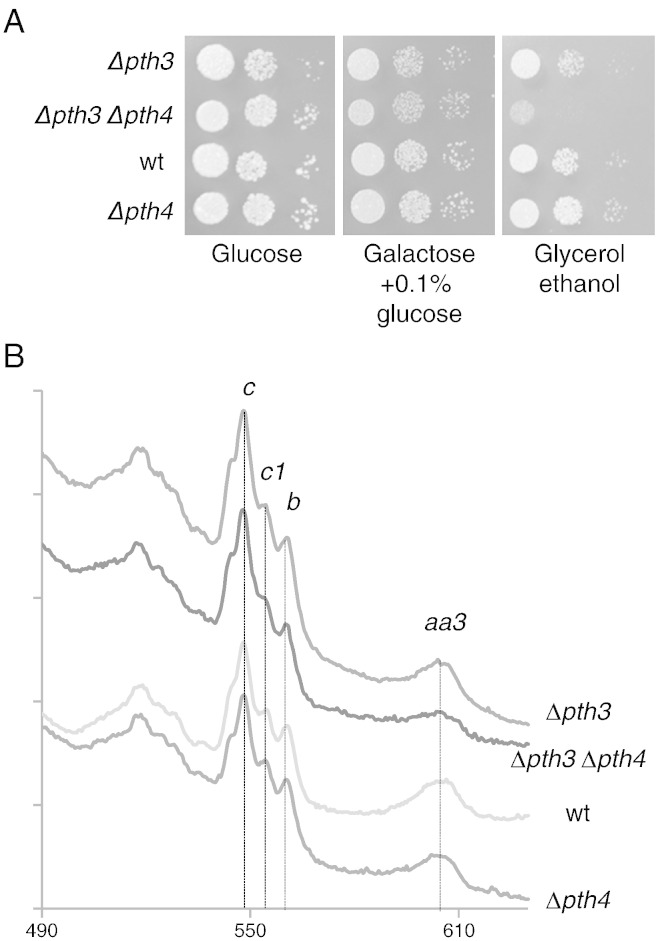
Phenotypic and spectral analysis of the *pth* gene deletions. The growth and cytochrome spectra were analyzed for a tetratype issued from the cross between a ∆*pth3* and a ∆*pth4* mutant. A. Serial ten-fold dilutions of overnight cultures were spotted onto complete medium containing the indicated carbon sources. Galactose and glycerol are both used to reveal respiratory growth defects in *S. pombe*. Photographs were taken after 5 (glucose), 8 (galactose) and 10 (glycerol/ethanol) days. B. Cells grown for 2 days on complete glucose medium were used to record low-temperature cytochrome spectra after fully reducing the cytochromes with dithionite. The peaks corresponding to cytochrome *aa_3_*, *b*, *c_1_* and *c* are indicated.

To determine if Pth3 and Pth4 have an important role in mitochondrial biogenesis the corresponding genes were deleted, the double mutant ∆*pth3*, ∆*pth4* was constructed and the effect of the mutations on respiratory growth was examined. In *S. pombe*, an inability to grow on galactose indicates a strong respiratory deficiency (Chiron et al., 2007). Fig. 3A shows that all the deletion strains were able to grow on galactose. On glycerol/ethanol medium, the deletion of *pth4* had no effect, but the deletion of *pth3* led to a slight reduction in growth, while the double deletion showed a clear reduction in respiratory growth. Low temperature cytochrome spectra of cell pastes (Fig. 3B) showed that the ∆*pth4* strain had a wild type cytochrome spectrum, the ∆*pth3* strain showed a very slight diminution in the level of cytochrome *aa3* and this was accentuated in the ∆*pth3*, ∆*pth4* double mutant. Taken together, these results show that Pth3 and Pth4 are not essential for respiratory growth, but they do have partially overlapping roles in mitochondrial biogenesis, which are more visible on complex IV.

### 3.3 Interactions between pth3 and pth4, and mrf1

In *S. pombe*, the deletion of *mrf1* does not lead to a complete respiratory deficiency (Soleimanpour-Lichaei et al., 2007), so we decided to see if Pth3 or Pth4 had some functional overlap with Mrf1. To do this we looked at the effect on respiratory growth of deleting or over-expressing *pth3* and *pth4* in a ∆*mrf1* background.

**Fig. 4:**
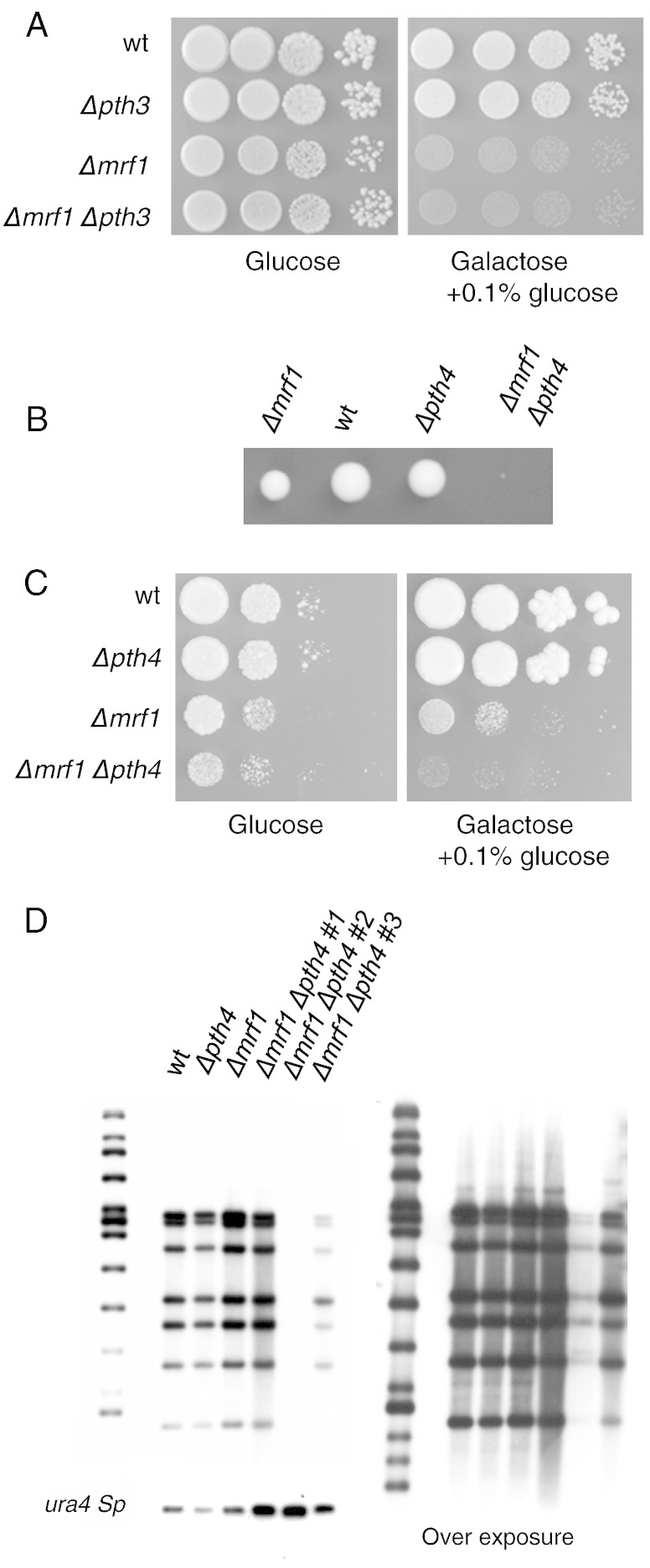
Interactions between *pth3* and *pth4*, and *mrf1*. ∆*mrf1* cells were crossed to ∆*pth3* and ∆*pth4* cells and tetrads were dissected to isolate double mutants. The ∆*mrf1* and ∆*pth4* cross was performed in the *ptp1*-*1* background, which allows the loss of the mitochondrial DNA. A. Growth of serial 10 fold dilutions of the wt, single mutants and double mutants on glucose and galactose media. B. Photograph of the germination of a tetrad corresponding to a tetratype. C. Serial ten-fold dilutions of cultures from the tetrad in B were spotted on complete medium containing glucose or galactose as main carbon source. D. Southern blot analysis of various *ptp1*-*1* strains: wild type, single mutations and three ∆*mrf1*, ∆*pth4* double mutations. Genomic DNAs were extracted, digested with *Hin*DIII and hybridized successively with the full mitochondrial DNA cloned in pBR322, or with the nuclear gene, *ura4*. The right panel corresponds to an overexposure of the blot probed with the mitochondrial DNA. The DNA marker (Raoul I, Appligène, Strasbourg, France) hybridizes with pBR322 sequences. Sizes of the detected mtDNA fragments are the following: 4314, 4077, 3403, 2455, 2114, 1651 and 1156 bp. The *Hin*DIII *ura4* gene fragment is 1.8 kb long.

The ∆*pth3*, ∆*mrf1* double mutant was constructed by crossing the two single mutants and has the same respiratory phenotype as the ∆*mrf1* mutant (Fig. 4A). However, when the ∆*pth4* and ∆*mrf1* strains were crossed, no viable double mutants were obtained; whenever ∆*pth4*, ∆*mrf1* double mutants were expected in a tetrad, we observed micro-colonies that were unable to grow further, suggesting that ∆*pth4* and ∆*mrf1* are co-lethal. This phenotype was reminiscent of the deletion of the mitochondrial elongation factor mtEF-Tu, which is essential for mitochondrial translation (Chiron et al., 2005). To circumvent this problem, we introduced the ∆*pth4* and ∆*mrf1* mutations into a *ptp1*-*1* background, which allows *S. pombe* to survive the loss of its mitochondrial genome (*rho^0^*) and therefore a loss of mitochondrial translation (Haffter and Fox, 1992). The results in Fig. 4B and C show that in the *ptp1-1* background we could obtain ∆*pth4*, ∆*mrf1* double mutants, they showed a delayed germination, very slow growth on glucose and an absence of growth on galactose.

In *S. cerevisiae* it is well known that mitochondrial translation is essential for the maintenance of the mitochondrial genome (reviewed in Contamine and Picard, 2000), and in *S. pombe ptp1-1* strains lacking mtEF-Tu show a strong depletion in their mitochondrial genome (Chiron et al., 2005). We decided to use Southern blotting to examine the level of the mitochondrial DNA in three independent ∆*pth4*, ∆*mrf1* double mutants in the *ptp1-1* background, and they were compared to the isogenic wild type and the single mutants. Fig. 4D shows that the three ∆*pth4*, ∆*mrf1* double mutants present different levels of mitochondrial DNA, varying from essentially wild type to almost undetectable, suggesting that the ∆*pth4*, ∆*mrf1* double mutation leads to a depletion in the mitochondrial DNA and that the three samples are at different stages of depletion. Whatever the level of depletion, the growth phenotypes of all the double mutants are identical to that shown in Fig. 4C.

**Fig. 5:**
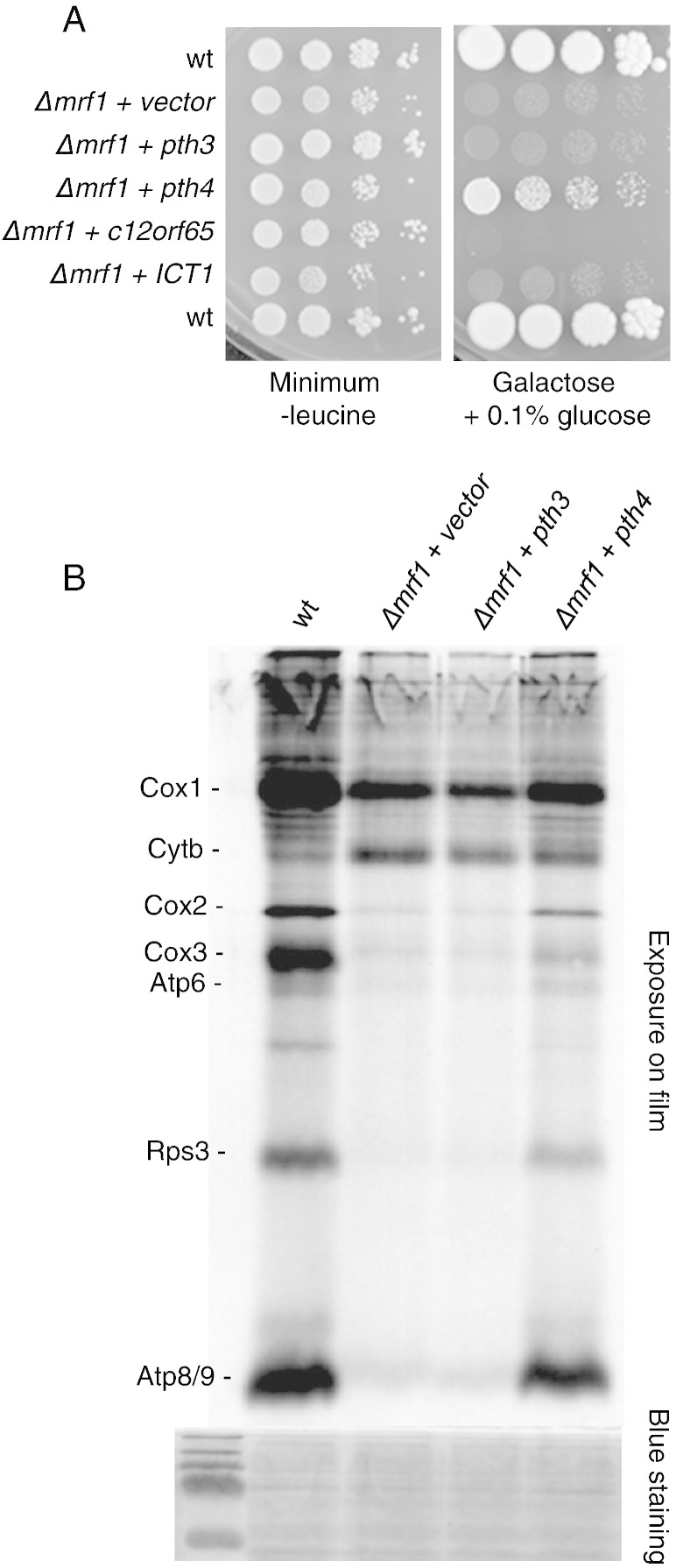
*Pth4* is a high copy suppressor of *Δmrf1*. A. ∆*mrf1* cells were transformed with the *S. pombe pth3*, or *pth4* cloned in pDUAL-FFH1, giving rise to proteins that are tagged FLAG_2_His_6_, or their human sequence homologs *C12orf65* or *ICT1* that also incorporated a FLAG tag, or with the empty vector pSC49. The growth of the transformants was compared to that of the wild type by spotting ten-fold serial dilutions of each strain onto minimal medium lacking leucine or complete galactose medium supplemented with 0.1% glucose. The plates were incubated for 7 and 10 days respectively. B. Mitochondrial proteins from the first four strains of panel A were labeled with [^35^S] cysteine and methionine in the presence of cycloheximide and separated on a 16% polyacrylamide gel. An autoradiograph of the full dried gel is presented, as well as a portion of the Coomassie blue stained gel to serve as a loading control.

As the deletion of *pth4* considerably exacerbates the ∆*mrf1* phenotype, we decided to determine if over-expression of *pth3* or *pth4* could improve the respiratory competence of the ∆*mrf1* strain. From the results in Fig. 5A, it is clear that over-expression of *pth3* has no effect on the ∆*mrf1* phenotype, but over-expression of *pth4* significantly improves the respiratory growth of the ∆*mrf1* strain. To find out if this effect was due to a modification of mitochondrial translation, we looked at de novo mitochondrial protein synthesis in the same strains, as described in the Materials and methods, and the results are shown in Fig. 5B. For reasons that are unclear, neo-synthesized Cytb is often low in wild type *S*. *pombe* strains (Kühl et al., 2011). These results show that with the exception of Cytb, the deletion of *mrf1* causes a severe reduction in all the de novo synthesized mitochondrial proteins. This pattern of synthesis is not affected by the over-expression of *pth3*, but the over-expression of *pth4* significantly increases the neo-synthesis of all the mitochondrial translation products, compared to the ∆*mrf1* strain.

Taken together, the results in Fig. 4, Fig. 5 show that there is an overlap of function between *pth4* and *mrf1* and that this affects the level of the de novo synthesis of the mitochondrial proteins.

### 3.4 Association of the Pth proteins with the ribosome

**Fig. 6:**
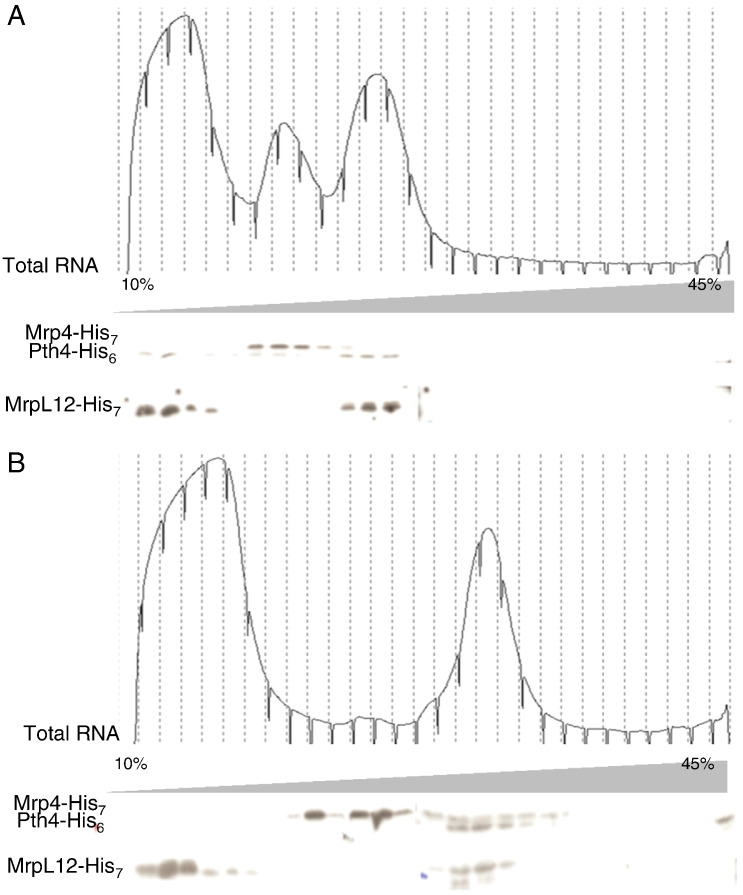
Sucrose gradient analysis of Pth4 in whole cell extracts. Whole cell extracts from the strain LD64-1 producing Pth4-His_6_, as well as Mrp4-His_7_ and MrpL12-His_7_ were made in the presence of EDTA, which favors ribosome dissociation (A) and in the presence of 50 mM MgCl_2_, which favors subunit association (B). These extracts were layered onto 10 to 45% sucrose gradients. Thirty fractions were collected after centrifugation. The absorbance trace at 254 nm, corresponding to total RNA is shown, as well as a western blot analysis of the fractions with an anti-His_6_ epitope antibody, which can recognize all three tagged proteins. Note that a longer exposure is generally needed to detect MrpL12-His_7_.

It is well known that release factors interact transiently with the large subunit of the ribosome (for a review see Youngman et al., 2008). Considering the interactions between *pth3* and *pth4*, and *pth4* and *mrf1* we decided to investigate whether the Pth proteins are associated with the mitochondrial ribosome. To do this triple tagged versions of *pth3* and *pth4* (YFP-FLAG-His_6_) under the control of the thiamine repressible promoter *nmt1* were integrated into CHP060-2D (*mrp4*-*His_7_*, *mrpl12*-*His_7_*) at the *leu1 locus* as described in the Materials and methods. Whole cell extracts were fractionated on sucrose gradients (10–45%) in the presence of EDTA, which will favor the dissociation of the ribosomal subunits, or MgCl_2_, at concentrations that will stabilize the assembled ribosome. Samples were collected and western blots were probed with anti-His_6_ antibodies to reveal Pth4 as well as Mrp4 and MrpL12, which are markers of the small and large ribosomal subunits respectively. As the experiments were performed on whole cell extracts, the absorbance peaks at 254 nm are due to the cytosolic rRNAs, which are far more abundant than the mitochondrial rRNAs; however, because of the level of resolution of the gradients we would expect the cytosolic and mitochondrial rRNAs to be present in essentially the same fractions, as indicated by the ribosomal marker proteins. In the presence of EDTA, we do not detect any assembled ribosomes and Pth4 co-sediments with the large ribosomal subunit, indicated by the presence of MrpL12. In the presence of MgCl_2_, we see some unassembled small ribosomal subunits, no unassembled large subunits and assembled ribosomes, indicated by the position of Mrp4 and MrpL12; in this case, Pth4 co-sediments with the assembled ribosomes (Fig. 6). This experiment was repeated using a strain where *mrf1* was deleted and Pth4 still co-sedimented with the assembled ribosomes (Fig. S2). Similar results were obtained for Pth3 in a wild type background (Fig. S3). Thus both Pth3 and Pth4 co-sediment with the large ribosomal subunit and assembled ribosomes and at least in the case of Pth4, this is not dependent on the presence of Mrf1, suggesting that like release factors, they are associated with the large ribosomal subunit.

### 3.5 Are the functions of the Pth proteins conserved through evolution?

In humans there are orthologs of Pth3 and Pth4, termed C12orf65 and ICT1 respectively. Initially, we decided to see if C12orf65 and ICT1 could alleviate the phenotype of the ∆*mrf1* mutant, to do this each ORF was fused to the *N*. *crassa* F0-ATPase subunit 9 presequence and cloned into a yeast expression vector and then transformed into a ∆*mrf1* strain. The results in Fig. 5A show that expression from either construct was unable to improve the respiratory growth of the ∆*mrf1* strain.

Next we decided to determine if Pth4 is able to associate with the human mitochondrial ribosome. For this, the *pth4* ORF was cloned with, or without the *N. crassa* F0-ATPase subunit 9 presequence and with a C-terminal FLAG epitope, into an integration vector that was used to transfect tetracycline inducible HEK293T cells. The cells carrying these constructs were tested for the expression of the FLAG epitope. Cells transfected with the construct lacking the *N. crassa* F0-ATPase subunit 9 presequence showed a very low level of Pth4-FLAG expression and no mitochondrial import, whereas cells carrying the construct with the *N. crassa* F0-ATPase subunit 9 presequence showed a high level of Pth4-FLAG expression. A fraction of this Pth4-FLAG protein was imported into mitochondria and the size of the detected protein suggested that the presequence had been cleaved (see Fig. S4).

**Fig. 7:**
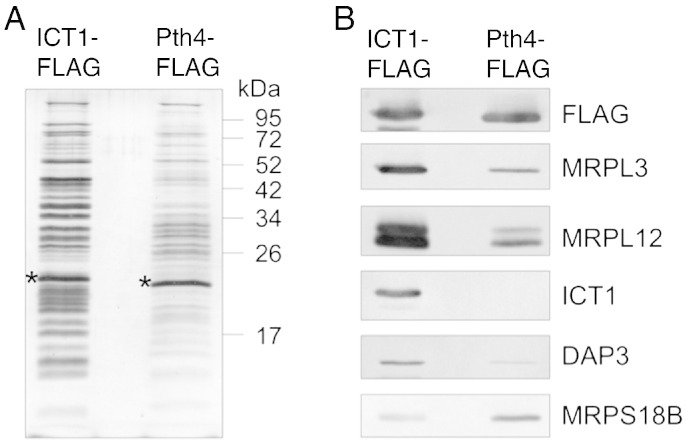
*S. pombe* Pth4-FLAG is able to immunoprecipitate a subset of human mitoribosomal proteins. HEK293T-ICT1-FLAG and HEK293T-Pth4-FLAG cell lines were induced for 3 days with 1 μg/ml tetracycline. Mitochondria were purified and lysed, the lysates were then used for immunoprecipitation via the FLAG tag. 10% of the elution fractions were analyzed by PAGE and western blot. A. Silver staining of the 15% PAGE of the elution fractions, * indicates the FLAG tagged protein. B. Western blot analysis using antibodies against ICT1 and other large subunit ribosomal (MRPL3, MRPL12) or small subunit (DAP3 and MRPS18B) ribosomal proteins from human mitochondria was performed to determine the relative level of ribosomal proteins immunoprecipitated by Pth4-FLAG and ICT1-FLAG. To verify the presence of the FLAG tagged protein in each elution fraction FLAG antibodies were also applied.

The cell line expressing Pth4-FLAG was used to determine if the protein was associated with the human mitochondrial ribosome. Transfected HEK293T cells were induced for three days to express either the Pth4-FLAG, or ICT1-FLAG, mitochondria were purified and treated with DNase 1 and proteinase K to minimize unspecific contamination from cytosolic proteins. The mitochondria were lysed and immunoprecipitations performed with anti-FLAG antibodies; the eluates were then analyzed by PAGE, silver staining and western blot. The results in Fig. 7A show different elution patterns for ICT1 control versus Pth4-FLAG immunoprecipitate, where there are fewer and less proteins. When examined by western blot (Fig. 7B) the levels of the FLAG tagged proteins were similar in both eluates allowing semi-quantitative comparisons of co-immunoprecipitating proteins to be made. The large ribosomal subunit proteins MRPL3 and MRPL12, and the small ribosomal subunit protein DAP3 were detected but at lower levels than in the ICT1 control IP. MRPS18B was present but at slightly elevated levels in the Pth4-FLAG eluate while the endogenous ICT1 was not detectable. This suggests that Pth4 from *S. pombe* can interact with human mitoribosomal proteins, but that the interaction is weaker and different to that of the endogenous ICT1.

**Fig. 8:**
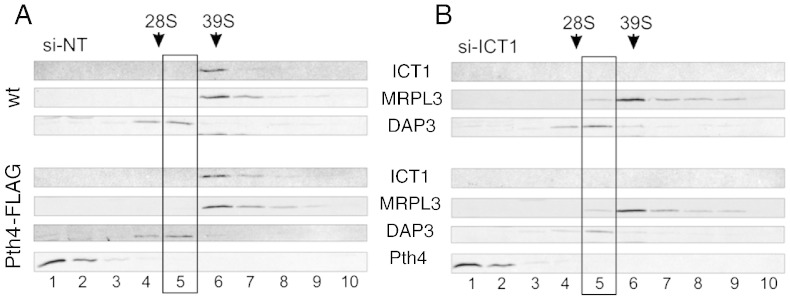
Pth4-FLAG cannot suppress the mitoribosomal defect caused by ICT1 depletion. Both HEK293T-WT and HEK293T-Pth4-FLAG cells were treated with si-NT (A), or si-ICT1 (B) for 3 days. At the same time the cultures were treated with tetracycline to induce the FLAG tagged protein. Cells were then harvested and lysed. Lysates (700 μg) were separated on 10–30% isokinetic sucrose gradients. The fractions 1–10 were analyzed by western blotting using anti-ICT1 antibodies to confirm ICT1 depletion in the siRNA targeted samples. The ribosomal profile in each sample was determined using anti-MRPL3 and anti-DAP3 antibodies and the distribution of Pth4-FLAG was determined by using anti-FLAG antibodies.

In human mitochondria, when ICT1 is depleted there is a conformational change in the 39S large ribosomal subunit, resulting in a shift of MRPL3 (a protein of the large ribosomal subunit) from fractions 6/7, to 5/6 in sucrose gradients (Richter et al., 2010). To determine if Pth4-FLAG can suppress this phenotype, HEK293T-wt and HEK293T-Pth4-FLAG cells were treated with either a control (si-NT) or an ICT1 (si-ICT1) siRNAs for three days, with concomitant tetracycline treatment to induce Pth4 expression in the relevant cell line. Cells were then harvested and lysed, and 700 μg of each sample separated on 10–30% isokinetic sucrose gradients. The isolated fractions were analyzed by western blot. In the HEK293T-wt cells treated with si-NT (Fig. 8A, upper panel), ICT1 was present principally in fraction 6, co-sedimenting with MRPL3 (fractions 6/7), whereas DAP3, a member of the small ribosomal subunit was present in fractions 4 and 5. The si-NT treated HEK293T-Pth4-FLAG cells showed a similar result (Fig. 8A, lower panel). Upon depletion of ICT1 (Fig. 8B), ICT1 was no longer detectable and MRPL3 was now detected in fraction 5, with no difference between the HEK293T-wt and HEK293T-Pth4-FLAG cells (Fig. 8B, upper/lower panels); thus Pth4-FLAG is not able to suppress the ICT1 depletion phenotype. Significantly, in both experiments Pth4-FLAG was mainly detected in fractions 1–3 and not fractions 5 or 6, consistent with the previous data suggesting that Pth4-FLAG is not associated with the large ribosomal subunit, even when mitoribosomes are depleted of ICT1.

## 4 Discussion

The orderly termination of translation is a key step in the quality control and overall process of protein synthesis. In the *S. pombe* mitochondrial system, homology searches have identified a single class I release factor, Mrf1, but deletion of the corresponding gene only leads to a partial respiratory deficiency (Soleimanpour-Lichaei et al., 2007 and Fig. 4B). At present, it has not been definitively demonstrated that Mrf1 is a release factor, but we have shown that it is important for mitochondrial translation (Fig. 5B) and Soleimanpour-Lichaei et al. (2007) have shown that human mtRF1a, which recognizes the stop codons UAA and UAG, is able to complement the ∆*mrf1* mutation in both *S. cerevisiae* and *S. pombe*. Thus, the evidence strongly suggests that Mrf1 is a mitochondrial release factor; however, the partial phenotype of the ∆*mfr1* strain implies that least one other protein can also act as a release factor.

Class I release factors are essentially tRNA mimics (Moffat and Tate, 1994) with conserved GGQ and decoding motifs (tip of α-5 helix and PXT) that interact with the same centers in the A site of the ribosome as the CCA and anticodon loop of the tRNA (see Youngman et al., 2008 for a review). Pth3 and 4 also contain the conserved GGQ motif and modeling of this domain indicates that it has a similar structure to the equivalent domain in Mrf1 (shown for Pth4 in Fig. S5). However, both proteins are considerably smaller than Mrf1 and notably do not contain an equivalent to either the α-5 helix or the PXT motif found in Mrf1. We have undertaken a preliminary investigation of Pth3 and 4 and their possible functional overlap with Mrf1.

We have shown that Pth3 and Pth4 are both mitochondrial proteins that co-sediment with the large ribosomal subunit and the assembled ribosome in yeast. The deletion of *pth4* gives no obvious phenotype, while the deletion of *pth3* gives a weak respiratory phenotype that is exacerbated in the double deletion strain. The respiratory phenotype of the ∆*pth3*, ∆*pth4* double mutant is however still relatively mild (see Fig. 4). Although it is tempting to speculate that this indicates some sort of functional overlap between Pth3 and Pth4, in reality, this is far from clear considering that the phenotype is weak and that over-expression of *pth4* in the ∆*pth3* strain does not improve the respiratory phenotype (data not shown).

The situation is more straightforward when we examine the interactions between the *pth* genes and *mrf1*. Our results give no indication of an interaction between *pth3* and *mrf1*; however, it is clear that there is some interaction between *pth4* and *mrf1*. Over-expression of *pth4* in a ∆*mfr1* strain increases the level of de novo translation and results in considerably improved respiratory growth, while deletion of *pth4* significantly exacerbates the ∆*mfr1* phenotype. In a wild type background the ∆*pth4*, ∆*mrf1* double mutant is essentially unviable, and in a *ptp1*-*1* background it undergoes depletion of mitochondrial DNA (Fig. 4, Fig. 5), characteristic of impaired mitochondrial translation (Chiron et al., 2005). Thus, we conclude that in the absence of Mfr1, Pth4 is able to act as a release factor.

In humans, C12orf65 and ICT1 are orthologs of Pth3 and Pth4 respectively, and both have been shown to be important for mitochondrial translation. Depletion of C12orf65 reduces complex IV activity by about 50% but has a less dramatic effect than depletion of ICT1 (Kogure et al., 2012). ICT1 has been shown to be a constituent of the large ribosomal subunit and intact mitoribosomes, with a codon independent Pth activity (Richter et al., 2010). We have shown that when expressed in *S. pombe* neither C12orf65 nor ICT1 can complement the respiratory deficiency of a ∆*mrf1* strain (the phenotype of the ∆*pth3* strain is not sufficiently strong to allow meaningful complementation experiments). Further, when Pth4 is expressed in HEK293T cells, in contrast to control FLAG IP with mitochondrially targeted luciferase (Richter et al., 2010, Rorbach et al., 2008), it is able to immunoprecipitate (IP) a number of mitoribosomal proteins, although not endogenous ICT1. This together with the gradient data suggest that the interaction of Pth4 with the human mitochondrial ribosome is not the same as ICT1 that integrates into the mitoribosome, and is probably via access to the free A-site. This is consistent with the inability to suppress the conformation change that occurs in the large ribosomal subunit upon ICT1 depletion (Fig. 7, Fig. 8). Pth4 therefore cannot functionally replace ICT1.

In *S. cerevisiae*, Mrf1 is essential for mitochondrial translation and the maintenance of the mitochondrial genome even though *S. cerevisiae* contains a sequence homolog of Pth4 (Yol114c). The reason for this difference between *S. cerevisiae* on the one hand and *S. pombe* and man, on the other is not clear. A possible explanation is that *S. cerevisiae* mitochondrial mRNAs have long 3′ UTRs, while both *S. pombe* and man have very short, or nonexistent 3′ UTRs. In *S. pombe* the mature 3′ ends of the mitochondrial transcripts are produced by Pah1 and Par1, in the absence of either of these proteins, mitochondrial mRNAs with long unprocessed 3′ UTRs accumulate (Hoffmann et al., 2008). Interestingly, ∆*pah1* and ∆*mrf1* are essentially co-lethal (Fig. S6), which could suggest that the presence of a long 3′ UTR reduces the effectiveness of Pth4.

We have shown that the over-expression of *pth4* can partially suppress the phenotypes associated with the deletion of *mrf1*. Multi-copy suppressors can act in a variety of ways, either indirect or direct; for example, an excess of Pth4 could enhance the activity of another protein that compensates for the absence of Mrf1. However, considering the co-sedimentation of Pth4 with the large subunit of the mt ribosome and its structural similarity to Mrf1, we think it's more probable that Pth4 is able to act as a release factor, at least in the absence of mrf1, but it is not clear if this is part of the normal role of Pth4 in the wild type cell. While Pth3, Pth4 and Mrf1 all appear to be able to associate with the large ribosomal subunit as part of the fully assembled ribosome, it is not known whether different factors can be present on the same ribosome, or if the different factors define subpopulations of “different” mitoribosomes. Further experiments will be necessary to clarify the roles of the different release factors.
