# Supplementary material for: Interactions between peptidyl tRNA hydrolase homologs and the ribosomal release factor Mrf1 in S. pombe mitochondria
Source: Mitochondrion. 2013 Nov;13(6):871–80. doi: 10.1016/j.mito.2013.07.115 (PMC3919214; doi:10.1016/j.mito.2013.07.115)
Supplement: Supplementary file 1 — Supplementary material. [file mmc1.pdf]

Figure S1 Multiple alignment of the full-length sequences of Mrf1, Pth3 and Pth4 from *S. pombe* and their orthologs from *S. cerevisiae* and humans

```
SpMrf1        1 ----------MLLTKKVLWYFRHGIEYQIRSIACK---KGYSEHNVSRVLIEKARSLSSE
ScMrf1        1 MWLSKFQFPSRSIFKGVFLGHKLPLLVRLTSTTTNSKSNGSIPTQYTELSPLLVKQAEKY
HsmtRF1a      1 ----------------------------MRSRVLWGAARWLWPRRAVGPARRPLSSGSPP
SpPth3        1 ------------------------------------------------------------
ScPth3        1 ------------------------------------------------------------
HsICT1        1 ------------------------------------------------------------
SpPth4        1 ------------------------------------------------------------
ScPth4        1 ------------------------------------------------------------
HsC12orf65    1 ------------------------------------------------------------

SpMrf1       48 YLQFHQVNNKDQSAMTNDTDLAKRIARLRRVHNAYSKFKSLSEQISDLKQMEAQESDAEV
ScMrf1       61 EAELKDLDKDLSCGIHFDVNKQKHYAKLSALTDTFIEYKEKLNELKSLQEMIVS--DPSL
HsmtRF1a     33 LEELFTRGGPLRTFLERQAGSEAHLKVRRPELLAVIKLLNEKERELRETEHLLHDENEDL
SpPth3        1 ------------------------------------------------------------
ScPth3        1 ------------------------------------------------------------
HsICT1        1 ------------------------------------------------------------
SpPth4        1 ------------------------------------------------------------
ScPth4        1 ------------------------------------------------------------
HsC12orf65    1 ------------------------------------------------------------

SpMrf1      108 KMMAVTEINEISNKIPKSIEDLENTLLPQADSYALPALIEIRPGVGGTEAAIFANELVEM
ScMrf1      119 RAEAEQEYAELVPQYETTSSRLVNKLLPPHPFADKPSLLELRPGVGGIEAMIFTQNLLDM
HsmtRF1a     93 RKLAENEITLCQKEITQLKHQIILLLVPSEETDENDLILEVTAGVGGQEAMLFTSEIFDM
SpPth3        1 ------------------------------------------------------------
ScPth3        1 ------------------------------------------------------------
HsICT1        1 ------------------------------------------------------------
SpPth4        1 ------------------------------------------------------------
ScPth4        1 ------------------------------------------------------------
HsC12orf65    1 ------------------------------------------------------------

SpMrf1      168 YFQYANFKGWNCKFISKSAVQGLEAITEAIFSIEGEGAYGHLMLEGGVHRVQRTPATETK
ScMrf1      179 YIGYANYRKWKYRIISKNENESGSGIIDAILSIEEAGSYDRLRFEAGVHRVQRIPSTETK
HsmtRF1a    153 YQQYAAFKRWHFETLEYFPSELG-GLRHASASIGGSEAYRHMKFEGGVHRVQRVPKTEKQ
SpPth3        1 --------------------------------------------MLCAARKCLNKTFISV
ScPth3        1 -------------------------------------------------------MMRGA
HsICT1        1 -------------------MAATRCLRWGLSRAGVWLLPPPARCPRRALHKQKDGTEFKS
SpPth4        1 -----------------------------------------------MFANFRNCFKIKN
ScPth4        1 ---------------------------MTTLMGKFKLTGRSPLFVLQPMLHCKKQQFVEE
HsC12orf65    1 -----------------------------------MSTVGLFHFPTPLTRICPAPWGLRL

SpMrf1      228 GRVHTSTASVIVLPQVSN------DESSSLYDSSEVKIEVMRSRGAGGQHVNRTESAVRL
ScMrf1      239 GRTHTSTAAVVVLPQIGDESAKSIDAYERTFKPGEIRVDIMRASGKGGQHVNTTDSAVRL
HsmtRF1a    212 GRVHTSTMTVAILPQPTEIN--------LVINPKDLRIDTKRASGAGGQHVNTTDSAVRI
SpPth3       17 RLNAFSCLAELNFRHTWYCSKKETPYQLERLQEEDIEETFICGKGPGGQKINKTSIVAQV
ScPth3        6 SKRSISSAAVLLIKKNKLPPR---PKFTPEMEAQCTEKFLHGGRGPGGQKINKCNSKVQL
HsICT1       42 IYSLDKLYPESQGSDTAWRVPNGAKQADSDIPLDRLTISYCRSSGPGGQNVNKVNSKAEV
SpPth4       14 SRLIYDNINKCLLTKEETNQLLKFIHLKWKPAKDQVQISFSRSSGPGGQNVNKLNTKVIV
ScPth4       34 AVRLISNKKIGKKSDFVQARNWVGALNVTGLPLNQFILRYDRASGPGGQNVNKVNSKCTL
HsC12orf65   26 WEKLTLLSPGIAVTPVQMAGKKD-YPALLSLDENELEEQFVKGHGPGGQATNKTSNCVVL

SpMrf1      282 THIPTGITVSMQDSRSQHQNKEKAFLVLNSRLAALNAAKENEAERLKRKNQVTSSDRSEK
ScMrf1      299 THIPSGIVVSMQDERSQHKNKAKAFTILRARLAEKERLEKEEKERKARKSQVSSTNRSDK
HsmtRF1a    264 VHLPTGVVSECQQERSQLKNKELAMTKLRAKLYSMHLEEEINKRQNARKIQIGSKGRSEK
SpPth3       77 KHIPTGIIVRSQDTRSREQNRCIARKRLTEKVDEFKHGNDSLLARKVQRIVKKKKQREKK
ScPth3       63 RHEPTGIVVECQETRSREQNRKLARLKLARELAASYDTMPSREEALLQWHRQ--QKRSQR
HsICT1      102 RFHLATAEWIAEPVRQKIAGELILTSESSRYQFRNLADCLQKIRDMITEASQTPKEPTKE
SpPth4       74 NLPFKQLESCIPMFLINHFKTCEMLRNYRIQNGIKIYSQKTRSQHKNIEDALNKISDLIN396
ScPth4       94 TLSGLSNCAWIPQEVRNILSSGRFRYYAKGSDSIVIQSDETRSRETN--KLKCFEKLVQE413
HsC12orf65   85 KHIPSGIVVKCHQTRSVDQNRKLARKILQEKVDVFYNGENSPVHKEKREAAKKKQERKKK380
                                                                             162
                                                                             155
SpMrf1      342 LRTYNFNQNRVTDHRIGLSMHDLSTFMQGE---EKFDEFLEKIRIWNREQLLLHSEIV 196
ScMrf1      359 IRTYNFPQNRITDHRCGFTLLDLPGVLSGERL-DEVIEAMSKYDSTERAKELLESN-- 182
HsmtRF1a    324 IRTYNFPQNRVTDHRINKTLHDLETFMQGDYLLDELVQSLKEYADYESLVEIISQKV- 202
SpPth3      137 SKRKYGNKIDDQDSSLNNNEAKQDVI-------------------------------- 166
ScPth3      121 RRSVAKYEQREEAARVEKEEREARDREMVRELFRR-----------------------
HsICT1      162 DVKLHRIRIENMNRERLRQKRIHSAVKTSRRVDMD-----------------------
SpPth4      134 KSAETLYVPDTPPEKIARISILKKESNEKRLSEKKYKQKKKTQRRITMD---------
ScPth4      152 IRQTCQFPNDTTAETSKKWNKIKEKANKERLLDKKVHSDKKKNRSKIKFNY-------
HsC12orf65  145 AKETLEKKKLLKELWESSKKVH------------------------------------
```

The alignment of the nine full-length sequences was made using the ClustalW and Boxshade programs from the http://www.ch.embnet.org server with the following parameters: opening gap penalty and end gap penalty: 10; extending gap penalty: 2; separation gap penalty: 5. The full-length sequences are shown. *S pombe* peptidyl-tRNA hydrolases are SpPth3: Spbc1105.18c; SpPth4: Spac589.11. *S. cerevisiae* peptidyl-tRNA hydrolases are ScPth3: Ylr281c ; ScPth4: Yol114c.

Figure S2 Sucrose gradient analysis of Pth4 in the absence of Mrf1

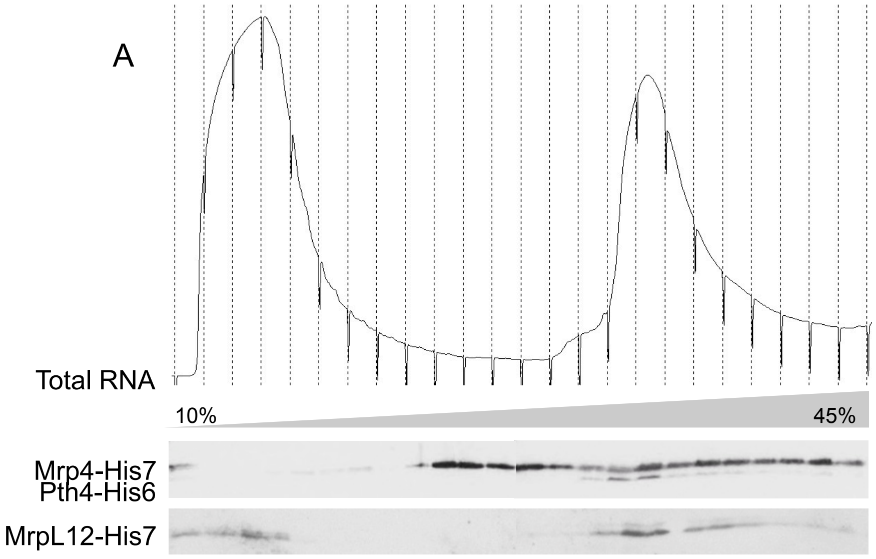

NB329-1 (h-, *ade6M-216, leu1-32, ura4D-18, his3Δ, Δmrf1::kan*^{R}) was crossed to CHP060-2D (h$^+$, *ade6M, leu1-32, mrps4-His*$_7$*::kan*^{R}*, mrpl12-His*$_7$*::kan*^{R}) and dissected to give CHP072-5D (h-, *ade6M, leu1-32, his3Δ, mrps4-His*$_7$*::kan*^{R}*, mrpl12-His*$_7$*::kan*^{R}*, Δmrf1::G418*).

This was then crossed to LD64 (*h*$^+$*, ade6, mrp4-His*$_7$*::kan*^{R}*, mrpl12-His*$_7$*::kan*^{R}*, leu1::pth4-FLAG*$_2$*-His*$_6$) and dissected to give CHP079-6C (*h?, ade6M, mrps4-His*$_7$*::kan*^{R}*, mrpl12-His*$_7$*::G418, Δmrf1::kan*^{R}*, leu1::pth4-FLAG*$_2$*-His*$_6$).

hole cell extracts of CHP079-6C were made in the presence of 50 mM $MgCl_2$, which favors subunit association. This extract was layered onto 10 to 45 % sucrose gradient and after centrifugation thirty fractions were collected, of which 24 were analyzed. The absorbance trace at 254 nm, corresponding to total RNA is shown. A western blot analysis is presented under the OD trace. It was performed using an anti-His$_6$ epitope antibody that can recognize all three tagged proteins. A longer exposure is generally needed to detect MrpL12-His$_7$.

Figure S3 Sucrose gradient analysis of Pth3 in whole cell extracts

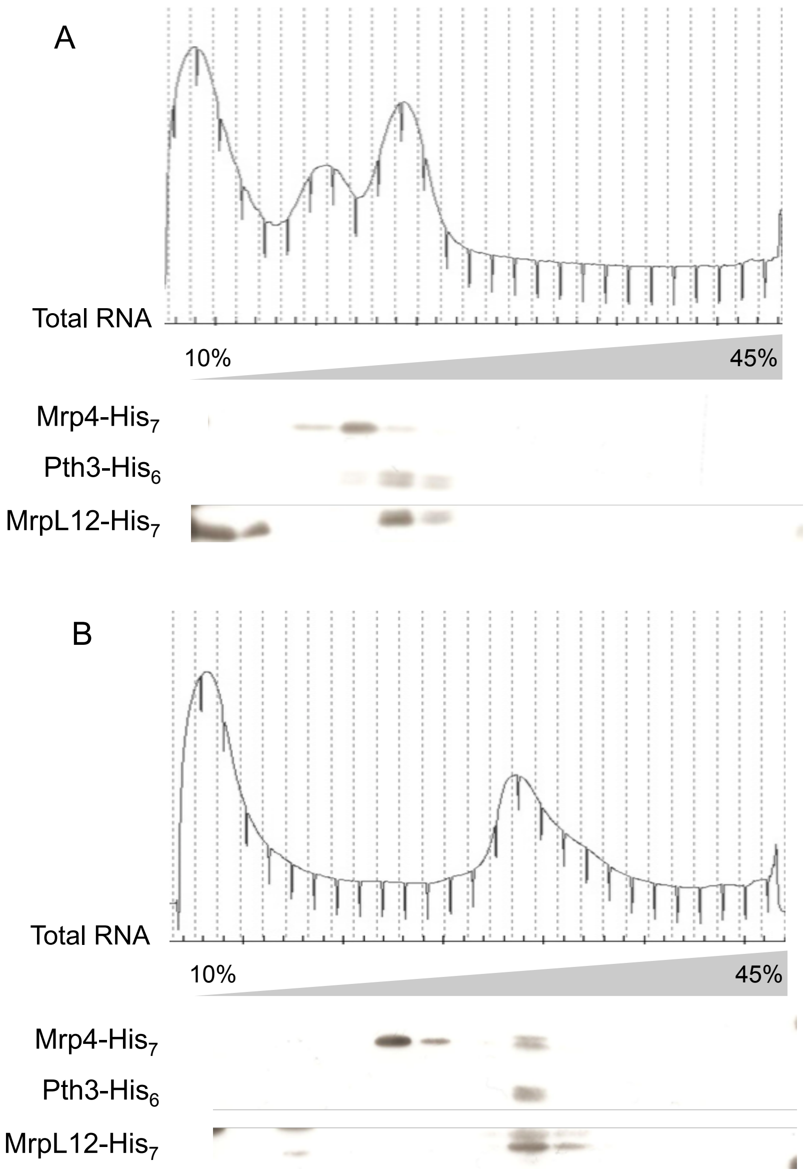

Whole cell extracts from the strain LD63-1 producing Pth3-His_6 protein, as well as Mrp4-His_7 and MrpL12-His_7 were made in the presence of EDTA, which favors ribosome dissociation (**A**) and in the presence of 50 mM $MgCl_2$, which favors subunit association (**B**). These extracts were layered onto 10 to 45 % sucrose gradients. Thirty fractions were collected after centrifugation. The absorbance trace at 254 nm, corresponding to total RNA is shown. A western blot analysis is presented under the OD trace. It was performed on alternate fractions using an anti-His_6 epitope antibody that can recognize all three tagged proteins. Note that a longer exposure is generally needed to detect MrpL12-His_7.

Figure S4 Cellular localization of Pth4-FLAG within human HEK293T cells

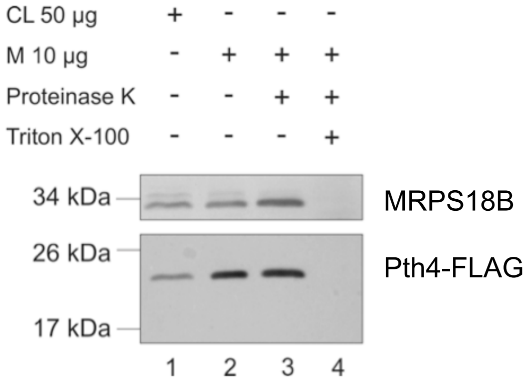

Expression of the protein corresponding to the *N. crassa* F0-ATPase subunit 9 presequence fused to Pth4-FLAG, was induced in HEK293T cells over 3 days. Cell lysate (50 μg, lane 1) and isolated mitochondria (10 μg), untreated (lane 2) or treated with proteinase K, in the absence (lane 3) or presence of 1% Triton X-100 (lane 4) were analyzed by western blot. Pth4-FLAG was identified using anti-FLAG antibodies, and antibodies against MRPS18B, a member of the human mitoribosomal small subunit, were used as a positive control. The expected molecular weight of the Pth4-FLAG fusion protein is 29 kDa, the observed molecular weight is ~21.5 kDa, suggesting that the N-terminal presequence has been removed by proteolytic cleavage.

Figure S5 Comparison of the predicted structures of Mrf1 and Pth4

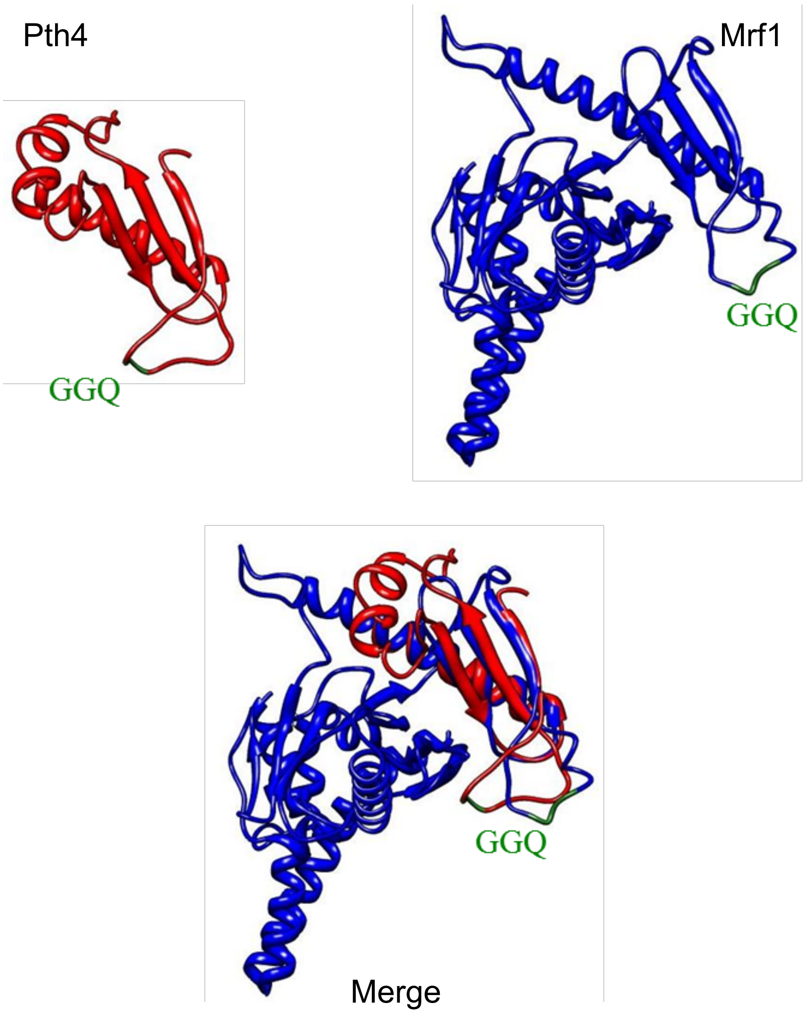

Predicted structures for residues 68 to 385 from Mrf1 (blue) and 47 to 137 from Pth4 (red) were retrieved from the Modbase databank under accession numbers Q09691 and Q9HDZ3 respectively. Beta sheets are indicated as arrows and alpha helices as helical structures, the conserved GGQ motif is in green. When the structures are merged, residues 285 to 321 of Mrf1 overlap residues 77 to 137 of Pth4. The C-terminal region of Pth4 appears unstructured in all predictions, thus to maintain clarity we have used partial sequences for the construction of the models.

Figure S6 Interaction between *mrf1* and *pah1*

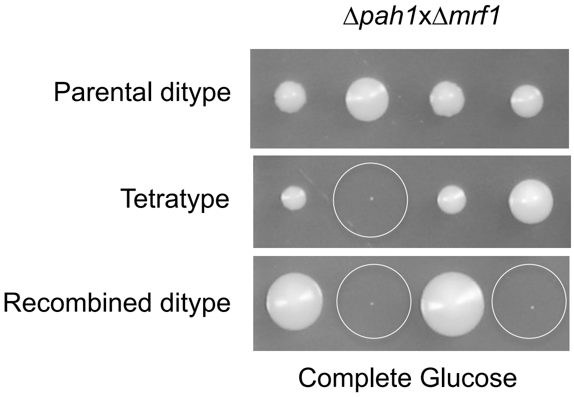

Δ*mrf1* (NB349-3A) and Δ*pah1* (PAH53M3) cells were crossed and photographs of tetrads were taken after a 15-day incubation on complete glucose medium; double mutants (inferred from the analysis of the growing spores) produce micro-colonies, which are indicated by white circles.
